# The loss-of-function disease-mutation G301R in the Na^+^/K^+^-ATPase α_2_ isoform decreases lesion volume and improves functional outcome after acute spinal cord injury in mice

**DOI:** 10.1186/s12868-017-0385-9

**Published:** 2017-09-08

**Authors:** Ditte Gry Ellman, Toke Jost Isaksen, Minna Christiansen Lund, Safinaz Dursun, Martin Wirenfeldt, Louise Helskov Jørgensen, Karin Lykke-Hartmann, Kate Lykke Lambertsen

**Affiliations:** 1 Neurobiology Research, Institute of Molecular Medicine, University of Southern Denmark, 5000 Odense C, Denmark; 2 Department of Biomedicine, Aarhus University, 8000 Aarhus C, Denmark; 3 Department of Clinical Medicine, Aarhus University, 8000 Aarhus C, Denmark; 4 Centre for Membrane Pumps in Cells and Disease-PUMPKIN, Danish National Research Foundation, Aarhus University, 8000 Aarhus C, Denmark; 5 Department of Pathology, University of Southern Denmark/Odense University Hospital, Odense, 5000 Odense C, Denmark; 6 Department of Clinical Research, University of Southern Denmark/Odense University Hospital, Odense, 5000 Odense C, Denmark; 7 Department of Clinical Genetics, Aarhus University Hospital, 8000 Aarhus C, Denmark; 8 Department of Neurology, Odense University Hospital, 5000 Odense C, Denmark; 9 BRIDGE, Inter-Disciplinary Guided Excellence, Department of Clinical Research, University of Southern Denmark, 5000 Odense C, Denmark

**Keywords:** Spinal cord injury, Na^+^/K^+^-ATPase α_2_ isoform, Functional recovery, Reduced lesion volume

## Abstract

**Background:**

The Na^+^/K^+^-ATPases are transmembrane ion pumps important for maintenance of ion gradients across the plasma membrane that serve to support multiple cellular functions, such as membrane potentials, regulation of cellular volume and pH, and co-transport of signaling transmitters in all animal cells. The α_2_Na^+^/K^+^-ATPase subunit isoform is predominantly expressed in astrocytes, which us the sharp Na^+^-gradient maintained by the sodium pump necessary for astroglial metabolism. Prolonged ischemia induces an elevation of [Na^+^]_i_, decreased ATP levels and intracellular pH owing to anaerobic metabolism and lactate accumulation. During ischemia, Na^+^/K^+^-ATPase-related functions will naturally increase the energy demand of the Na^+^/K^+^-ATPase ion pump. However, the role of the α_2_Na^+^/K^+^-ATPase in contusion injury to the spinal cord remains unknown. We used mice heterozygous mice for the loss-of-function disease-mutation G301R in the *Atp1a2* gene (α_2_^+/G301R^) to study the effect of reduced α_2_Na^+^/K^+^-ATPase expression in a moderate contusion spinal cord injury (SCI) model.

**Results:**

We found that α_2_^+/G301R^ mice display significantly improved functional recovery and decreased lesion volume compared to littermate controls (α_2_^+/+^) 7 days after SCI. The protein level of the α_1_ isoform was significantly increased, in contrast to the α_3_ isoform that significantly decreased 3 days after SCI in both α_2_^+/G301R^ and α_2_^+/+^ mice. The level of the α_2_ isoform was significantly decreased in α_2_^+/G301R^ mice both under naïve conditions and 3 days after SCI compared to α_2_^+/+^ mice. We found no differences in astroglial aquaporin 4 levels and no changes in the expression of chemokines (CCL2, CCL5 and CXCL1) and cytokines (TNF, IL-6, IL-1β, IL-10 and IL-5) between genotypes, just as no apparent differences were observed in location and activation of CD45 and F4/80 positive microglia and infiltrating leukocytes.

**Conclusion:**

Our proof of concept study demonstrates that reduced expression of the α_2_ isoform in the spinal cord is protective following SCI. Importantly, the BMS and lesion volume were assessed at 7 days after SCI, and longer time points after SCI were not evaluated. However, the α_2_ isoform is a potential possible target of therapeutic strategies for the treatment of SCI.

## Background

Spinal cord injury (SCI) results in massive cell loss at the site of the lesion. Injury to the central nervous system (CNS) causes a range of cellular and molecular changes that make changes to the local environment and impede regeneration. Cytokines and chemokines are released at the lesion site serving to recruit peripheral leukocytes to the injury site [1]. Astrocytes are the most numerous cells in the CNS and become reactive in response to neuronal injury.

In the intact spinal cord there is limited proliferation of astrocytes. However, in response to injury, inflammatory cytokines cause adult astrocytes to proliferate and give rise to reactive astrocytes [2, 3].

Astrocytes are important for maintaining homeostasis within the CNS including regulating ion concentrations, neurotransmitter levels, pH and water homeostasis, support of the blood–brain barrier, regulating synaptic formation and function, and neuronal support. After neuronal activity, astrocytes are essential for clearing extracellular K^+^ and regulating synaptic concentrations of the neurotransmitter glutamate. This is accomplished through the Na^+^-dependent glutamate transporters EAAT1 and EAAT2 [4]. The uptake of glutamate via glutamate transporters is forwarded by the Na^+^-gradient. Astrocytes, therefore, rely on Na^+^/K^+^-adenosine triphosphatase (Na^+^/K^+^-ATPase) to pump out accumulated intracellular Na^+^ and import extracellular K^+^ and thereby, the Na^+^/K^+^-ATPase ion pump maintains the Na^+/^K^+^ gradient essential for all cells [5, 6].

The Na^+^/K^+^-ATPase-maintained electrochemical gradients of Na^+^ and K^+^ across the plasma membrane are prerequisites for electrical excitability and secondary transport. The minimum constellation of an active pump consists of an alpha (α) and a beta (β) subunit [7, 8]. The α subunit is responsible for the catalytic and pharmacological properties [9], whereas the β and optional γ subunits may have regulatory functions [10–12]. Different isoforms combine to form kinetically distinct complexes in different cells and tissues [13, 14]. In mammals, four distinct α isoforms (α_1_–α_4_) have been identified. The α_1_ subunit isoform is ubiquitously expressed, the α_2_ is expressed in the brain, heart and skeleton muscles, the α_3_ isoform is expressed in the brain and heart, while the α_4_ subunit isoform is expressed exclusively in testis [15, 16]. In the adult brain, the α_2_ isoform is primarily expressed in astrocytes (alongside the α_1_ isoform), where it is coupled to various transporters (glutamate transporters and Na^+^/Ca^2+^ exchanger) [17–19]. Specifically, the α_2_ isoform is essential for clearance of extracellular K^+^ and glutamate released into the synaptic cleft [18, 20–22].

Prolonged ischemia induces an elevation of [Na^+^]_i_, increasing the energy demand of Na^+^/K^+^ ATPase, reversal of the Na^+^/glutamate co-transporter, cell swelling and activates volume regulatory processes [23]. The Na^+^/K^+^ ATPase activity (along with energy production processes) alongside passive K^+^ uptake mechanisms are upregulated in gliotic tissue located outside a spinal cord lesion to enhance such homeostatic mechanisms [24]. Interestingly, a recent study showed that remote ischemic post-conditioning could attenuate focal cerebral ischemia/reperfusion injury, and the neuroprotective mechanism was related with the down-regulation of aquaporin 4 (AQP4) in astrocytes [25]. Moreover, AQP4 knock out mice demonstrate significantly reduced cerebral edema and improved neurological outcome following ischemic stroke [26].

Increasing evidence points towards the Na^+^/K^+^-ATPase in regulating signaling pathways, such as the membrane-associated non-receptor tyrosine kinase Src, activation of Ras/Raf/ERK1/2, phosphate inositol 3-kinase (PI_3_K), PI_3_K-dependent protein kinase B, phospholipase C, [Ca^2+^]_i_ oscillations [27–29], and gene transcription (*Egr*-*1*, *Fos*, *June*, *Nr4a2*, *Hes1* and *Gabre*) [30].

Interestingly, increasing the Ca^2+^ concentration can modulate transcription in two ways; (1) by promoting translocation of nuclear factor-kappa B (NF-κB) from the cytosol to the nucleus, and (2) by phosphorylating the cAMP response element binding protein (CREB) [31].

To explore the role of the α_2_ isoform following contusion injury to the spinal cord, we took advantage of a newly generated knock-in mouse model harboring the Familial Hemiplegic migraine type 2 (FHM2) disease-related loss of function mutation G301R in the *Atp1a2* gene [22]. Heterozygous mice (α_2_^+/G301R^) display pathological relevant symptoms related to Familial Hemiplegic migraine type 2 (FHM2), and showed impaired glutamate uptake in in vitro-matured hippocampal mixed astrocyte-neuron cultures from α_2_^G301R/G301R^ E17 embryonic mice [22]. Moreover, NMDA-type glutamate receptor antagonists or progestin-only treatments reverted specific α_2_(+/G301R) behavioral phenotypes [22]. Mice homozygous for the G301R mutation (α_2_^G301R/G301R^) die immediately after birth [22]. In this study, SCI was performed on heterozygous α_2_^+/G301R^ mice to elaborate on the role of the α_2_Na^+^/K^+^-ATPase after SCI. We demonstrate that α_2_^+/G301R^ mice display significantly improved functional recovery and decreased lesion volume compared to littermate controls (α_2_^+/+^) already 7 days after SCI. Although long-term evaluations after SCI were not assessed, however, this study, suggests that decreasing the level of the α_2_ isoform might serve as a new potential therapeutic target in SCI treatment.

## Results

### Spinal cord of α_2_^+/G301R^ mice have reduced level of the α2 isoform

**Fig. 1:**
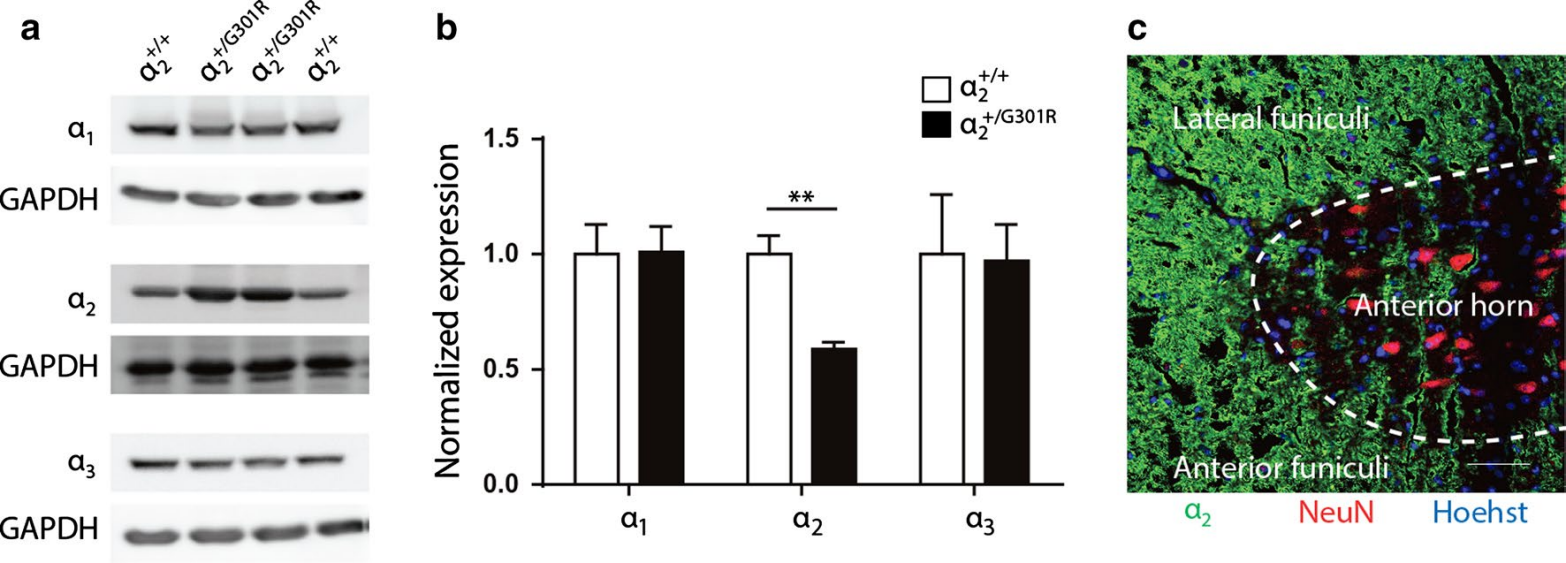
α_2_ isoform levels are reduced in α_2_^+/G301R^ compared to α_2_^+/+^ mice under naïve conditions. **a** Western blotting analysis showed comparable levels of α_1_ and α_3_, but reduced levels of α_2_ protein in the spinal cord of α_2_^+/G301R^ compared to α_2_^+/+^ mice under naïve conditions. **b** Quantification of α_1_, α_2_ and α_3_ isoform levels compared to GAPDH levels (Tukey’s multiple comparisons test; n.s (*p* = 0.99) for α_1_, **p < 0.01 (*p* = 0.0028) for α_2_ and n.s p = 0.99) for α_3_ (n = 4 mice/group). **c** Immunofluorescent staining of α_2_ (*green*) in a thoracic spinal cord section from naïve α_2_^+/G301R^ mice co-stained with the neuronal marker NeuN (*red, arrows*). *Scale bar* 50 μm

Previously, it was shown that the G301 mutation confers haploinsuffiency in heterozygous α_2_^+/G301R^ mice with reduced levels of the α_2_ isoform in various brain structures [22]. To investigate the α_2_ isoform levels in the spinal cord under naïve conditions, Western blotting was performed. As expected, α_1_ and α_3_ isoform levels were comparable between α_2_^+/G301R^ and α_2_^+/+^ mice, whereas α_2_ isoform levels were reduced approximately 40% in α_2_^+/G301R^ mice compared to littermates (Fig. 1a, b; Table 1). The morphology of the naïve spinal cord tissue was assessed by immunofluorescent detection of the α_2_ isoform and the neuronal marker Neuronal Nuclei (NeuN) and counterstained with Hoechst, and revealed no gross morphological differences between the α_2_^+/G301R^ and α_2_^+/+^ mice under naïve conditions (Fig. 1c, shown for a α_2_^+/G301R^ mouse only). In both α_2_^+/G301R^ and α_2_^+/+^ mice, the α_2_ isoform was preferentially detected in the white matter, both the posterior, lateral and anterior funiculi, with little astrocytic-localized α_2_ isoform in the grey matter.

**Table 1:** Quantification of α_1,_ α_2,_ and α_3_ isoform levels in the spinal cord in naïve conditions

	α_1_	α_2_	α_3_
α_2_^+/+^	1.00 ± 0.08	1.00 ± 0.05	1.00 ± 0.15
α_2_^+/G301R^	1.01 ± 0.06	0.59 ± 0.02	0.97 ± 0.09

Data are presented as mean ± SEM, n = 4/group. Data are normalized to GAPDH protein

### α_2_^+/G301R^ mice display improved functional outcome and decreased lesion volume 7 days after SCI

**Fig. 2:**
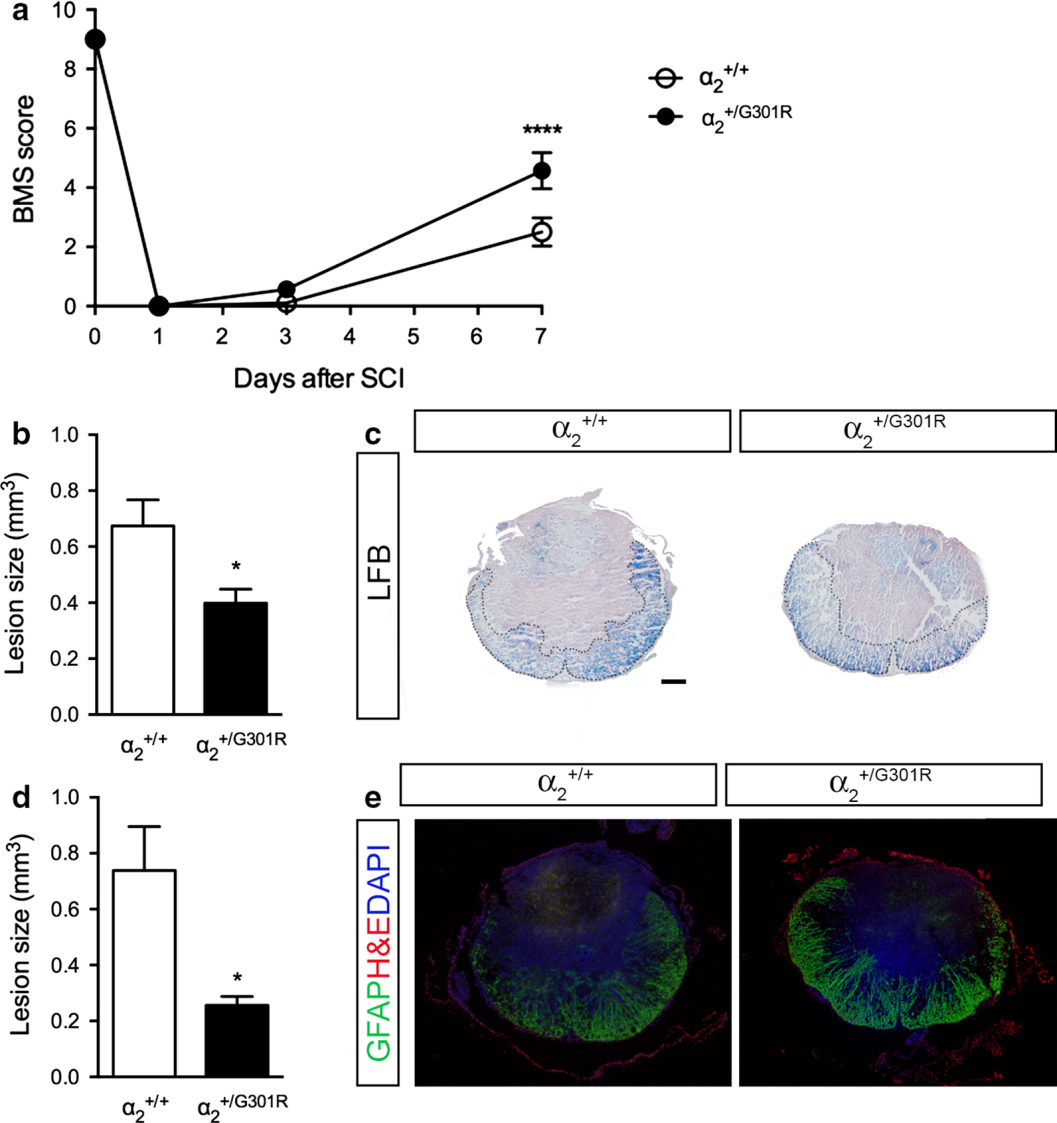
α_2_^+/G301R^ mice display decreased lesion size and improved functional recovery 7 days after SCI. **a** Functional outcome determined by BMS score in the open field test after SCI was significantly improved in α_2_^+/G301R^ compared to α_2_^+/+^ mice 7 days after SCI (two-way RM ANOVA: time ****p < 0.0001 F_3,42_ = 467; genotype *p < 0.05 F_1,14_ = 8.67; time:genotype **p < 0.01 F_3,42_ = 6.49, Bonferroni post hoc ****p < 0.0001, n = 7–9 mice/group). **b** Lesion volume was significantly decreased in α_2_^+/G301R^ compared to α_2_^+/+^ mice 7 days after SCI when analyzing luxol fast blue (LFB) stained sections (student’s *t* test, *p < 0.05, n = 6–8 mice/group). **c** Representative LFB stained thoracic spinal cord sections from α_2_^+/+^ and α_2_^+/G301R^ mice allowed 7 days survival after SCI. **d** Lesion volume was significantly decreased in α_2_^+/G301R^ compared to α_2_^+/+^ mice 7 days after SCI when analyzing GFAP/Nissl/DAPI stainings (student’s *t* test, *p < 0.05, n = 6–8 mice/group). **e** Representative GFAP/Nissl/DAPI stained thoracic spinal cord sections from α_2_^+/+^ and α_2_^+/G301R^ mice allowed 7 days survival after SCI. Results are expressed as mean ± SEM. *Scale bar* 200 μm

To date, the role of the Na^+^/K^+^-ATPase in SCI has not been addressed despite its essential role in astroglial functions. To examine the role of the astrocytic α_2_ isoform, we performed SCI on the α_2_^+/G301R^ mice and compared the effect to α_2_^+/+^ mice after 7 days in order to assess any correlations. The α_2_^+/G301R^ and α_2_^+/+^ mice were subjected to SCI and subsequently allowed 7 days post-surgical survival. The α_2_^+/G301R^ mice significantly improved their BMS score compared to α_2_^+/+^ mice (Fig. 2a), demonstrating that reducing the levels of the α_2_Na^+^/K^+^-ATPase in the spinal cord improved functional outcome after SCI in acute experiments (7 days post SCI). To evaluate the injury site, luxol fast blue (LFB) and glial fibrillary acidic protein (GFAP)/Nissl/DAPI staining was performed. Both analysis of LFB and GFAP/Nissl/DAPI stained sections revealed significantly reduced lesion volumes in α_2_^+/G301R^ mice compared to α_2_^+/+^ mice 7 days after SCI (Fig. 2b–d).

### Reduced α_2_ isoform levels after spinal cord injury in α_2_^+/G301R^ mice

**Fig. 3:**
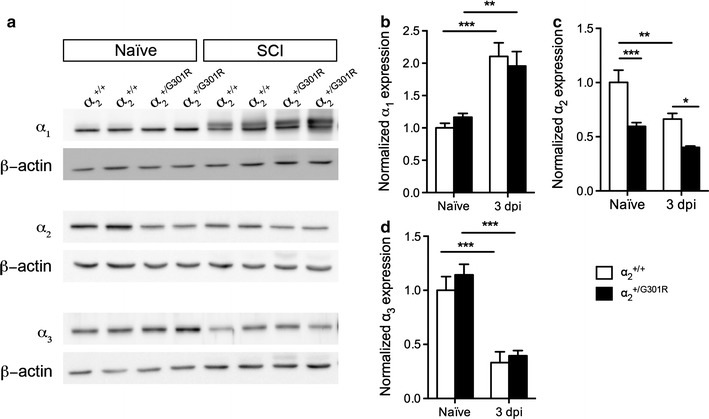
Changes in α_1_, α_2_, and α_3_ protein levels after SCI. **a** α_1_, α_2_, and α_3_ isoform levels in naïve conditions and 3 days after SCI were evaluated in spinal cord lysates from α_2_^+/+^ and α_2_^+/G301R^ mice by Western blot analysis. Data are normalized to β-actin protein expression. Representative experiments are shown. **b**–**d** Western blot quantification showed a significant increase in α_1_ isoform levels 3 days after SCI, with no difference between genotypes (two-way ANOVA: time ****p < 0.0001, F_1,12_ = 35.06, **p < 0.01) (**b**), a significant decrease in α_2_ isoform levels 3 days after SCI, with significantly reduced levels in α_2_^+/G301R^ compared to α_2_^+/+^ mice (two-way ANOVA: time ***p < 0.001, F_1,20_ = 16.88; genotype ****p < 0.0001, F_1,20_ = 26.67) (**c**), and a significant decrease in α_3_ isoform levels 3 days after SCI, with no difference between genotypes (two-way ANOVA: time ****p < 0.0001, F_1,12_ = 53.06) (**d**). Results, expressed as fold change in relation to naïve α_2_^+/+^ mice, are the mean ± SEM of 5 mice/group. ***p < 0.001, **p < 0.01, *p < 0.05, Bonferroni’s post hoc

In order to investigate whether the protein level of the α_2_ isoform was altered following SCI, Western blotting was performed on tissue from both naïve and SCI mice. Moreover, the protein levels of the two other CNS-expressed Na^+^/K^+^-ATPase α subunit isoforms, α_1_ and α_3_, were included (Fig. 3a). The α_1_ levels were significantly increased in both α_2_^+/+^ and α_2_^+/G301R^ mice 3 days after SCI compared to naïve conditions, with no difference between genotypes (Fig. 3b). In contrast, α_2_ levels were significantly decreased only in α_2_^+/+^ mice 3 days after SCI compared to naïve conditions, whereas no change was observed in α_2_ levels in the α_2_^+/G301R^ mice (Fig. 3c). Both under naïve conditions and 3 days after SCI, α_3_ levels were significantly decreased in α_2_^+/G301R^ compared to α_2_^+/+^ mice. The α_3_ levels were found to be significantly decreased after SCI compared to naïve conditions in both α_2_^+/+^ and α_2_^+/G301R^ mice, however no difference between the two genotypes was observed (Fig. 3d).

### Aquaporin 4 protein levels were comparable between α_2_^+/G301R^ and α_2_^+/+^ mice

**Fig. 4:**
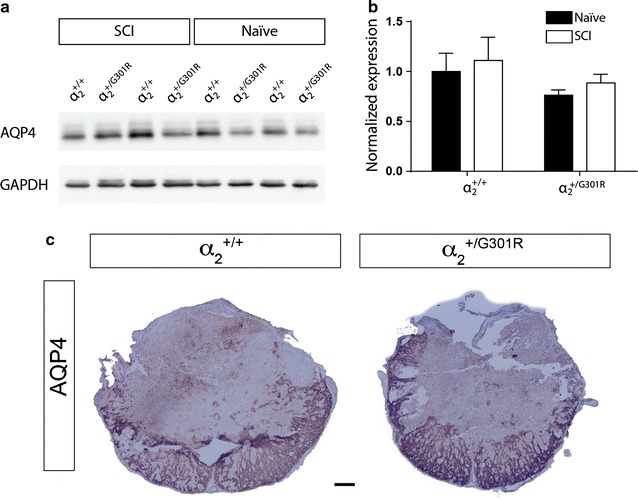
AQP4 protein levels in naïve and SCI-treated α_2_^+/+^ and α_2_^+/G301R^ mice. **a** AQP4 protein levels in naïve conditions and 3 days after SCI were evaluated in spinal cord lysates from α_2_^+/+^ and α_2_^+/G301R^ mice by Western blot analysis (**b**). Data are normalized to GAPDH protein expression, with no difference between genotypes or treatment (Tukey’s multiple comparisons test, naïve α_2_^+/+^ verus SCI-treated α_2_^+/+^, α_2_^+/G301R^ and naïve α_2_^+/G301R^ *p* = 0.96, *p* = 0.71 and *p* = 0.95, respectively, and SCI-treated α_2_^+/+^ versus naïve and SCI-treated α_2_^+/G301R^, *p* = 0.43 and *p* = 0.74, respectively, and naïve α_2_^+/G301R^ versus SCI-treated α_2_^+/G301R^, *p* = 0.94) (n = 4 animal/group). **c** Immunohistochemical staining for AQP4 was comparable between α_2_^+/+^ and α_2_^+/G301R^ mice 3 days after SCI. Analysis was based on five sections from each animal, n = 4 mice/group. *Scale bars* low magnification = 200 μm; high magnification = 50 μm

We next examined if levels of AQP4 was altered following SCI, in line with previous studies on cerebral edema [25, 26]. We performed Western blotting and immunohistochemistry on tissue from both naïve and SCI mice. The AQP4 levels were not significantly altered between genotypes, α_2_^+/+^ and α_2_^+/G301R^ mice, nor were the level significantly different between naïve conditions and SCI-treated animals (Fig. 4a, b; Table 2). Immunohistochemistry on spinal cord sections demonstrated that SCI induced loss of AQP4 staining within the lesion area and that the gross morphology of the AQP4 staining was comparable between α_2_^+/+^ and α_2_^+/G301R^ mice (Fig. 4c).

**Table 2:** Quantification of AQP4 protein levels in the spinal cord in naïve and SCI-treated conditions

	AQP4	
	Naïve	SCI
α_2_^+/+^	1 ± 0.37	1.11 ± 0.46
α_2_^+/G301R^	0.76 ± 0.11	0.89 ± 0.17

Data are presented as mean ± SEM, n = 4/group. Data are normalized to GAPDH protein

In summary, the levels of AQP4 protein were reduced after SCI, but no difference was observed between α_2_^+/+^ and α_2_^+/G301R^ mice after 7 days. Moreover, no differences in the overall gross distribution of AQP4 in spinal cord sections was not observed.

### Chemokine levels were comparable between α_2_^+/G301R^ and α_2_^+/+^ mice

**Fig. 5:**
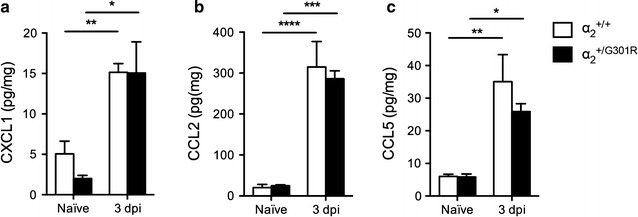
Chemokine expression profiling after SCI. **a** CXCL1 (two-way ANOVA: time ***p < 0.001, F_1,12_ = 28.75), **b** CLL2 (two-way ANOVA: time ****p < 0.0001, F_1,12_ = 71.50), and **c** CCL5 (two-way ANOVA: time ***p < 0.001, F_1,12_ = 31.76) protein levels were quantified by multiplex technology in naïve mice and 3 days after SCI in α_2_^+/G301R^ and α_2_^+/+^ mice. For each protein, results are expressed as mean ± SEM, n = 4 mice/group. ****p < 0.0001, ***p < 0.001, **p < 0.01, *p < 0.05, Bonferroni’s post hoc

In order to investigate whether changes in lesion size could be a consequence of changes in the inflammatory environment within the lesioned cord, multiplex analysis was used to investigate changes in chemokines. We found that CXCL1 (Fig. 5a), CCL2 (Fig. 5b), and CCL5 (Fig. 5c) protein levels were significantly upregulated in the lesioned spinal cord of α_2_^+/G301R^ and α_2_^+/+^ mice compared to naïve conditions; however, we observed no differences between the two genotypes at this time point.

### The inflammatory response was comparable between α_2_^+/G301R^ and α_2_^+/+^ mice 3 days after SCI

**Fig. 6:**
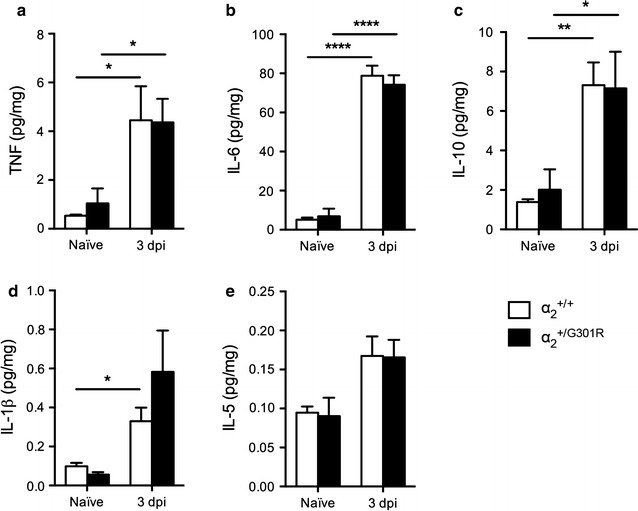
Cytokine expression profiling after SCI. **a** TNF (two-way ANOVA: time **p < 0.01, F_1,12_ = 16.1), **b** IL-6 (two-way ANOVA: time ****p < 0.0001, F_1,12_ = 302.90), **c** IL-10 (two-way ANOVA: time ***p < 0.001, F_1,12_ = 20.77), **d** IL-1β (two-way ANOVA: time **p < 0.01, F_1,12_ = 11.45), and **e** IL-5 protein levels were quantified by multiplex technology in naïve mice and 3 days after SCI in α_2_^+/G301R^ and α_2_^+/+^ mice. For each protein, results are expressed as mean ± SEM, n = 4 mice/group. ****p < 0.0001, **p < 0.01, *p < 0.05, Bonferroni’s post hoc

Using multiplex analysis, potential changes in cytokine expression were examined. We found that TNF (Fig. 6a), IL-6 (Fig. 6b) and IL-10 (Fig. 6c) were all significantly upregulated in the lesioned cord of α_2_^+/G301R^ and α_2_^+/+^ mice 3 days after SCI compared to naïve conditions, but no differences between genotypes were observed. In contrast, IL-1β was only significantly upregulated in α_2_^+/+^ mice 3 days after SCI compared to naïve conditions (Fig. 6d). No change was observed in IL-5 levels (Fig. 6e).

### Microglial/leukocyte activation is comparable between α_2_^+/G301R^ and α_2_^+/+^ mice 7 days after SCI

**Fig. 7:**
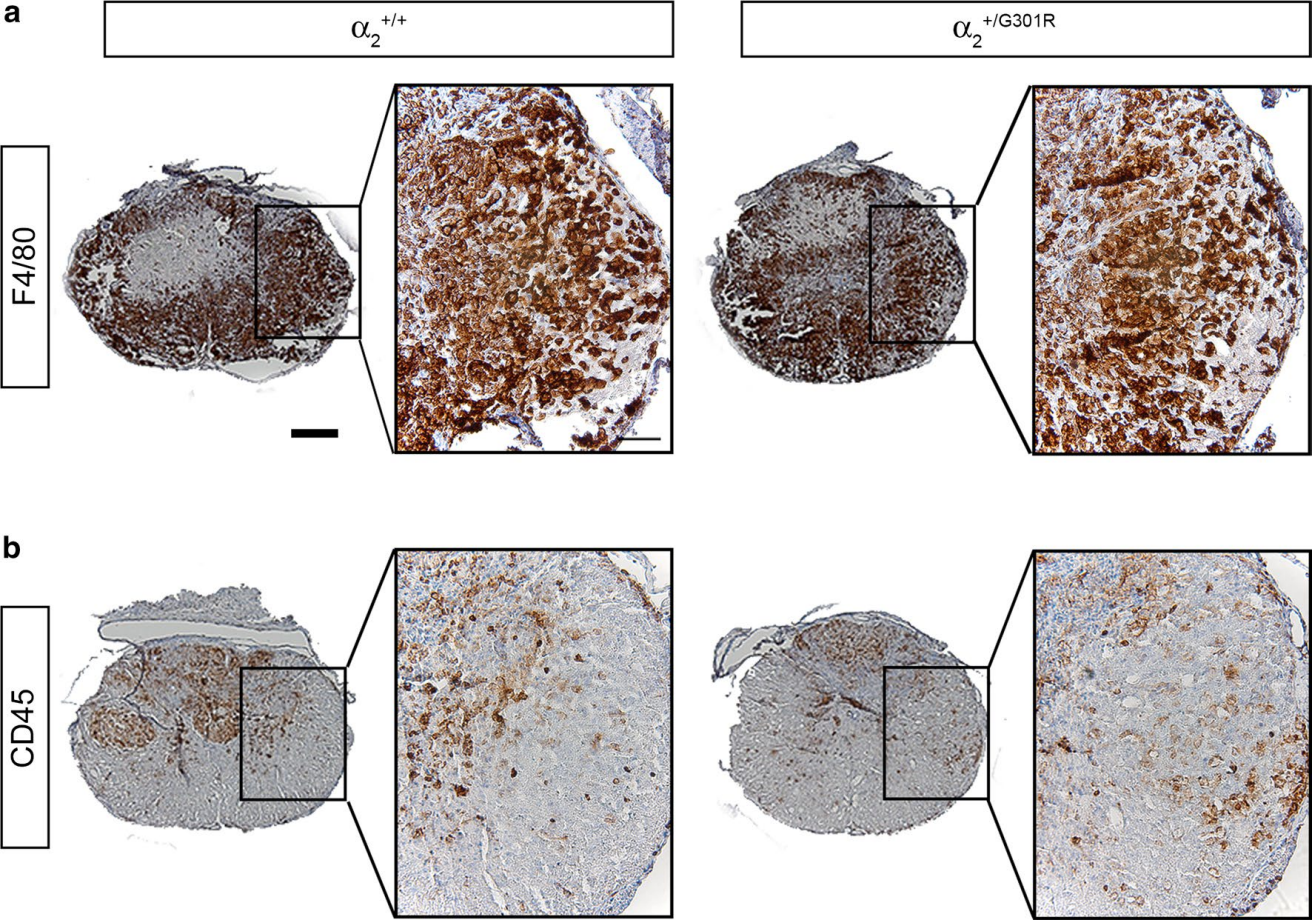
Microglial and leukocyte immunoreactivity 7 days after SCI. Immunohistochemical staining for F4/80 (**a**) and CD45 (**b**) were comparable between α_2_^+/+^ and α_2_^+/G301R^ mice 3 days after SCI. Analysis was based on 5 sections from each animal, n = 4 mice/group. *Scale bars* low magnification = 200 μm; high magnification = 50 μm

Macrophages can respond to endogenous stimuli that are rapidly generated following injury or infection [32]. These early stimuli can exert a marked (usually transient) effect on the physiology of macrophages. The response of macrophages to e.g. tissue damage can predict how these cells will respond during an adaptive immune response. We used the EGF-like module-containing mucin-like hormone receptor-like 1 (F4/80) glycoprotein and the CD45 antigen (CD stands for cluster of differentiation; also know as leukocyte common antigen (LCA)) as a specific cell-surface markers for microglia/macrophages [33] 7 days after SCI [34]. Based on microscopic evaluation, F4/80^+^ and CD45^+^ cells located near the epicenter displayed a macrophage-like morphology with large round cell bodies, whereas F4/80^+^ and CD45^+^ cells located further away from the epicenter displayed a more microglial-like morphology with small cell bodies and numerous branched processes. No apparent difference in the distribution or density of either F4/80^+^ (Fig. 7a) or CD45^+^ (Fig. 7b) cells between α_2_^+/+^ and α_2_^+/G301R^ mice was observed. All together, these data suggest that microglial and leukocyte activation and recruitment following SCI are not affected by reduced levels of the α_2_ isoform in the spinal cord.

## Discussion

Using a knock-in mouse model with the loss-of-function disease-mutation G301R in the *Atp1a2*-gene encoding the astrocyte-specific α_2_-isoform of the Na^+^/K^+^-ATPase [22, 35], we demonstrated that α_2_^+/G301R^ mice displayed decreased lesion volume and improved functional outcome 7 days after SCI, without altering the inflammatory response to SCI. The BMS and lesion volume were assessed 7 days after SCI, and evaluation at longer time points after SCI were not included. Moreover, the mechanisms underlying both acute and long term responses post SCI in the α_2_^+/G301R^ mice remain to be addressed.

The α_2_^+/G301R^ mice expressed reduced (~40%) α_2_ isoform levels in their spinal cords under naïve conditions. Interestingly, while both α_1_ and α_3_ isoform levels were significantly altered in both genotypes following moderate SCI, the α_2_ isoform was only significantly decreased in α_2_^+/+^ mice following SCI injury, whereas the α_2_ isoform level remained comparable between naïve and SCI-injured tissues in the α_2_^+/G301R^ mice. This might reflect the fact that there is a lower threshold for α_2_ isoform expression and cell survival in the α_2_^+/G301R^ mice.

After moderate SCI injury, the recovery was evaluated by functional outcome, estimation of lesion volumes and changes in pro- and anti-inflammatory cytokines and potential changes in microglial/leukocyte activation. Interestingly, the functional recovery of the α_2_^+/G301R^ mice was superior to α_2_^+/+^ mice and correlated with a significantly reduced lesion size in the α_2_^+/G301R^ compared to α_2_^+/+^ mice. In support of these findings, it was recently found that knock-down of the α_2_Na^+^/K^+^-ATPase in astrocytes deficient of the mutant superoxide dismutase 1 (SOD1) was able to protect motor neurons from degeneration in co-cultured primary motor neurons [36]. Heterozygous in vivo knock-down of the *Atp1a2* gene by lentiviral-mediated RNAi in the spinal cord of SOD1 mutant mice suppressed motor neuron degeneration and subsequently increased the life span of mutant SOD1 mice [36]. Moreover, in SOD-deficient astrocytes, mitochondrial respiration and inflammatory gene expressions were induced, suggesting that the upregulation of the α_2_Na^+^/K^+^-ATPase upregulated mRNAs encoding mitochondrial respiration and expression of secreted inflammatory factors (*Spp1*, *Lcn2*, *Clm1*, *Wnt1*, *Ccl11*, *Cxcl1*, *Ccr4*, *Il1Bbm*, *Itgb2*, *IIi1r1*) in SOD1 mutant astrocytes.

We interrogated the possibility that AQP4 might be regulated differentially in α_2_^+/G301R^ compared to α_2_^+/+^ mice. Overall, there was no difference in AQP4 level between the genotypes nor was there any significant difference between naïve and SCI-treated mice. Previous studies on cerebral edema found that reduced levels of AQP4 significantly improved the outcome [25, 26], which suggests that maybe the α_2_Na^+^/K^+^-ATPase works independently of AQP4 in the area tested, or maybe the effect would be more evident in later stages after SCI, which awaits future testing.

We found that chemokines CXCL1, CCL2 and CCL5 and cytokines TNF, IL-6 and IL-10 were significantly upregulated in the lesioned spinal cord of α_2_^+/G301R^ and α_2_^+/+^ mice compared to naïve conditions, however, we observed no statistical differences between the two genotypes. CCL2 [37], IL-6 [38, 39] and IL-10 [40] have previous been shown to be upregulated following SCI. Interstingly, there appeared to be a tendency towards genotype differences in the expression of CCL5 and IL-1β and future studies will be required to make a functional link between pump function and these two cytokines.

The pathophysiology of SCI involves a primary mechanical injury and a delayed secondary injury due to a number of proposed mechanisms including ischemia, abnormal intracellular shifts of ions including Na^+^, and excitotoxic cell death. A reduction or defect to extrude Na^+^ by the Na^+^/K^+^-ATPase pump as a consequence of the mutation introduced will result in increased intracellular Na^+^ levels, which might increase even further following SCI. In fact, increased intracellular Na^+^ concentration has been suggested to exacerbate the effects of compression trauma to the spinal cord in rats [41]. Furthermore, the collapse of transmembrane Na^+^ and K^+^ gradients following SCI is expected to induce a reversed operation of the Na^+^-dependent glutamate transporters, leading to glutamate efflux and subsequent activation of glutamate receptors causing substantial Ca^2+^-dependent injury [42]. It has previously been shown that mature neuronal and astrocytic co-cultures from α_2_^G301R/G301R^ mice display significantly reduced uptake of glutamate, and subsequently, increased glutamate levels [22]. It remains to be functionally tested if astrocytes in the spinal cord of α_2_^+/G301R^ mice will be altered.

The Na^+^/K^+^-ATPase can also act as a signal transducer and a NF-κB activator by interacting with neighboring membrane proteins and organized cytosolic cascades of signaling proteins [27, 43]. The activity of the Na^+^/K^+^-ATPase is modulated by glutamate by NMDA receptor-nitric oxide production, leading to activation of cyclic GMP-PKG [44, 45]. Interestingly, ouabain can induce a time- and dose-dependent activation of NF-κB and an upregulation of *Tnf*-*α*, *Il*-*1β*, and *Bdnf* mRNA levels [46].

Several other studies have correlated reduced Na^+^/K^+^-ATPase activity to SCI. A rat model of SCI induced by extradural compression of the cord resulted in a 50% decrease in the activity of synaptosomal Na^+^/K^+^-ATPase 30 min after the compression injury [47].

## Conclusion

In conclusion, the α_2_^+/G301R^ mice recover highly significantly after SCI compared to littermate α_2_^+/+^ control mice tested 7 days after SCI. The molecular mechanisms regarding this rapid recovery as a consequence of less Na^+^/K^+^-ATPase activity in astrocytes in relation to inflammatory responses remain to be elucidated, and these results serves as a proof of concept study and open promising potential towards therapeutic applications towards SCI.

## Methods

### Mice

Animals were housed in ventilated cages at a 12-h light/dark cycle, under controlled temperature and humidity, and free access to food and water. Mice were cared for in accordance with the protocols and guidelines approved by The Danish Animal Inspectorate under the Ministry of Food and Agriculture, Denmark (J. No. 2013-15-2934-00924 to KLL and J. No. 2013-15-2934-00815 to KLH); experiments performed in accordance with the ARRIVE guidelines, and all efforts were made to minimize pain and distress. All animal procedures were approved by Institutional Animal Care and Use Committee at the University of Aarhus and Southern Danish University, Denmark. All experiments were performed blinded.

### Genotyping

Heterozygous α_2_^+/G801R^ mice [22] were genotyped by High Resolution Melt analysis (Roche Lightcycler^®^ 96 Real-Time PCR System) using primers F-5′-ggatgagggacagaacgaag and R-5′-catggagatcgagcatttca (Sigma-Aldrich).

### Induction of spinal cord injury

A ketamine (100 mg/kg, VEDCO Inc)/xylazine (10 mg/kg, VEDCO Inc) cocktail was used to anaesthetize mice, and mice were laminectomized between vertebrae T8 and T10, and the impactor lowered at a pre-determined impact force resulting in an approximate displacement of 500 μm (moderate injury) [48]. Contusion injury was induced with the mouse Infinite Horizon-0400 SCI Contusion Device (Precision Systems and Instrumentation, LLC). Following SCI, mice were sutured and injected with saline to prevent dehydration and buprenorphine hydrochloride (0.001 mg/20 g Temgesic) four times at 8-h intervals, starting immediately prior to surgery. Individually, mice recovery in single cages, where their post-surgical health status was monitored during a 24–48 h recovery period, and then observed twice daily for activity level, respiratory rate and general physical condition. Manual bladder expression was performed twice a day and body weight was monitored. Mice received s.c. prophylactic injections of antibiotic gentamicin (40 mg/kg) to prevent urinary tract infections. No mice died during experiments.

### Functional outcome

#### Basso mouse scale

Functional post-SCI recovery of hind limb function was determined by scoring of the locomotor hindlimb performance in the open field using the Basso Mouse Scale (BMS) system, a 0 to 9 rating system designed specifically for the mouse [48]. Under observer-blinded conditions, mice were evaluated over a 4-min period 1, 3, and 7 days after SCI. Only mice with a score below 2 on day 1 were included in the study. Mice were handled and pre-trained in the open field before surgery to prevent fear and/or stress behaviors that could bias the locomotor assessment.

### Tissue processing

#### Histopathology and immunohistochemistry

Mice were deeply anaesthetized using an overdose of pentobarbital (200 mg/ml) containing lidocaine (20 mg/ml) and perfused through the left ventricle with cold 4% paraformaldehyde (PFA) in phosphate-buffered saline (PBS). Spinal cords were quickly removed and tissue segments containing the lesion area (1 cm centered on the lesion) were paraffin-embedded and cut into 10 parallel series of 15 μm thick microtome sections. Sections were stored at room temperature.

#### Klüver-Barrera Luxol Fast Blue staining for myelinated fibers

For evaluation of lesion pathology, one series of sections from each animal was stained in Luxol Fast Blue (LFB) (0.1% LFB in 95% ethanol (EtOH) and 0.05% acetic acid) at 60 °C for 12 h. Sections were rinsed in 96% EtOH and distilled H_2_O, immersed briefly in lithium carbonate (0.05% Li_2_CO_3_ in distilled water) and differentiated in 70% EtOH, before rinsed thoroughly in distilled H_2_O and immersed in 0.05% lithium carbonate to stop further differentiation. Sections were submitted to hematoxylin, rinsed in running tap water and immersed briefly in eosin solution. Finally, sections were rinsed in 70% EtOH, followed by 3× 99% EtOH, placed in 3× xylene prior to mounting with Depex. Paraffin embedded sections were deparaffinized 3× 3 min in xylene, 3× 2 min in 99% EtOH and 2× 2 min in 96% EtOH, before staining.

#### Immunohistochemical staining for CD45 and F4/80

Heat-induced antigen retrieval was done on the paraffin embedded sections by boiling the sections in Tris-EGTA buffer, pH 9.0 (CD45), TRS buffer (Target Retrieval Solution, DAKO) (F4/80), or TEG buffer, pH 9.0 (AQP4), first 15 min at 900W, then 9 min at 440W. The sections were allowed to cool in the buffer before blocked for endogenous peroxidase and biotin activity. Sections were then incubated with anti-CD45 (1:100; clone 30-F11 (Ly 5); BD Pharmingen), anti-F4/80 (1:100, AbD Serotec), or anti-AQP4 (1:1250; AQP-004, Alomone labs) antibodies and detected using biotinylated rabbit anti-rat IgG (DAKO) (CD45 and F4/80) or biotinylated donkey anti-rabbit IgG diluted 1:200 followed by ready-to-use anti-rabbit horse-radish perioxidase (HRP)-labelled polymer (EnVision + System, DAKO) with diaminobenzidin (DAB^+^) as chromogen (DAKO). Nuclei were counterstained using Mayer’s haemalum w/4.5% chloralhydrate. As negative control, the primary antibody was omitted to check for any unspecific reaction from the detection system. As positive control for antibody-specificity, the staining was tested using a mouse multi block containing several different tissues including lymphatic organs. The activation state of CD45^+^, and F4/80^+^ microglia and leukocytes and AQP4 expression were investigated in 5 sections (representing 750 μm spinal cord) from each animal centered on the lesion epicenter.

#### Immunofluorescent staining for glial fibrillary acidic protein (GFAP)

One series of sections from each animal was deparaffinized and rehydrated by placing the sections 3× 3 min in xylene, 3× 2 min in 99% EtOH, 2× 2 min in 96% EtOH, 2 min in 70% EtOH and finally 5 min in running tap water. The sections were demasked using TEG-buffer by placing the sections in warm TEG-buffer in a steamer for 15 min, then letting them cool for 15 min at room temperature before rinsing them for 15 min in running tap water. Sections were then rinsed 3× 15 min in tris-buffered saline (TBS) before they were pre-incubated with 10% fetal bovine serum (FBS) in TBS with 0.5% Triton X-100 for 30 min. Sections were incubated with Alexa Fluor^®^ 488-conjugated anti-GFAP (clone 131-17719, ThermoFischer Scientific) diluted 1:400 for 1 h at room temperature and hereafter over night at 4 °C. Next day, sections were placed at room temperature for 30 min before they were rinsed in TBS for 10 min and then in TBS with 0.1% Triton X-100 for 10 min. The sections were then stained with NeuroTrace^®^ 530/615 Red Fluorescent Nissl Stain (ThermoFischer Scientific) for 20 min, and further rinsed 2× 10 min in TBS before the sections were immersed in a TBS solution containing 10 μM diamidino-2-phenylindole (DAPI) for 10 min. The sections were shortly rinsed in distilled water before they were mounted with ProLong Diamond. Control reactions were performed by omitting the primary antibody or by substituting the primary antibody with Alexa Fluor^®^ 488 conjugated mouse IgG1κ (ThermoFischer Scientific). Sections were devoid of staining in the FITC imaging filter.

#### Double immunofluorescent staining for the a_2_ isoform and neuronal nuclei (NeuN)

Sections were prepared as desribed above for GFAP staining. Primary antibodies α2 (Merck Millipore) diluted 1:300 and NeuN (clone A60, Merck Millipore) diluted 1:300 [49] were applied in 1% donkey serum PBS with 0.02% Triton X-100 overnight at 4 °C. The following day, secondary labeling was perfomred with Alexa Fluor^®^ 488-conjugated donkey anti-rabbit antibody and Alexa Fluor^®^ 568-conjugated streptavidin (Life Technologies) diluted 1:350 in 1% donkey serum PBS with 0.025% Triton X-100 for 1 h at room temperature. Hoechst (1:10,000) (Life technologies) was used to counterstain the nuclei. Sections were then mounted with fluorescence mounting medium (Dako) and subsequently analysed on a LSM510 laser-scanning confocal microscope using a 40× C-Apochromat water immersion objective NA 1.2 (Carl Zeiss). Zen 2011 software (Carl Zeiss) was used for image capturing and analysis.

### Lesion volume estimation

The volume of the injury was determined from the area of every tenth section sampled by systematic uniform random sampling. Area of the lesion site was estimated as previously described [48]. Digital images were acquired using the 4× lens on an Olympus BX51 microscope fitted with an Olympus DP70 digital camera and a computerized specimen stage (Prior, Multicontrol 2000 MW). The Image J software was used to calculate the area of the injury on each section. These areas were summarized and multiplied by the section distance, resulting in an estimate of the total volume after dehydration and paraffin embedding.

### Western blotting

Whole spinal cord protein samples from α_2_^+/G301R^ and α_2_^+/+^ littermate mice exposed to SCI and allowed 3 days survival in addition to naïve α_2_^+/G301R^ and α_2_^+/+^ littermate mice were prepared as described [50]. Equal amounts of protein were separated by SDS-PAGE on 10–14% (α_1,_ α_2_, α_3_ and AQP4) gels and electro-blotted onto nitrocellulose membranes (Pharmacia-Amersham). Membranes were blocked in PBS with 5% skimmed milk and 0.5% Tween-20 and incubated with the following primary antibodies: anti-α_1_ diluted 1:2000 (clone a6f-c, Developmenal Studies Hybridoma Bank), anti-α_2_ diluted 1:1000 (Merck Millipore), anti-α_3_ diluted 1:1000 (Merck Millipore), anti-AQP4 diluted 1:1000 (AQP-004, Alomone labs) anti-GAPDH diluted 1:1000 (Abcam), or anti-β-Actin diluted 1:2000 (Sigma-Aldrich) overnight at 4 °C.

Next, membranes were incubated with HRP-conjugated secondary antibodies (swine anti-rabbit HRP diluted 1:2000 (Dako) or rabbit anti-mouse HRP diluted 1:2000 (Dako)) for 1 h at room temperature. Visualization of blots was done in a LAS 3000 imager (Fujifilm) with Amersham ECL Western Blotting Detection Kit (GE Healthcare). Post densitometric analysis and image processing of blots were performed in Image J.

### Multiplex analysis

To measure cytokine protein levels by the MSD Mouse Proinflammatory V-Plex Plus Kit (IFNγ, IL-1β, IL-2, IL-4, IL-5, IL-6, IL-10, IL-12p70, CXCL1, TNF; K15012C, Mesoscale) under naïve conditions and 3 days after SCI, we used a SECTOR Imager 6000 (Mesoscale Discovery, Rockville, USA) Plate Reader according to the manufacturer’s instructions. The same samples as those used for Western blotting were diluted two-fold in Diluent 41 prior to measurement and 50 µl dilution was loaded in each well. Data was analyzed using MSD Discovery Workbench software [50, 51].

### Statistical analysis

Comparisons were performed using repeated measures (RM) or regular two-way ANOVA followed by multiple *t* test analysis or Bonferroni post hoc, or by Student’s *t* test. Analyses were performed using Prism 4.0b software for Macintosh, (GraphPad Software). Statistical significance was established for *p* < 0.05.

## Abbreviations

AQP4: Aquaporin 4
CD45: Lymphocyte common antigen
CCL2: Chemokine (C–C motif) ligand 2
CCL5: Chemokine (C–C motif) ligand 5
CNS: Central nervous system
CXCL1: Chemokine (C-X-C motif) ligand 1
EGF-like module-containing mucin-like hormone receptor-like 1: F4/80
GFAP: Glial fibrillary acidic protein
Interleukin: IL
LFB: Luxol fast blue
Na^+^/K^+^-ATPase: Na^+^/K^+^-adenosine triphosphatase
NeuN: Neuronal Nuclei
TNF: Tumor necrosis factor
SCI: Spinal cord injury
